# On Differences between Deterministic and Stochastic Models of Chemical Reactions: Schlögl Solved with ZI-Closure

**DOI:** 10.3390/e20090678

**Published:** 2018-09-06

**Authors:** Michail Vlysidis, Yiannis N. Kaznessis

**Affiliations:** Department of Chemical Engineering and Materials Science, University of Minnesota, Minneapolis, MN 55455, USA

**Keywords:** closure scheme, maximum entropy, non-equilibrium steady state, Schlögl

## Abstract

Deterministic and stochastic models of chemical reaction kinetics can give starkly different results when the deterministic model exhibits more than one stable solution. For example, in the stochastic Schlögl model, the bimodal stationary probability distribution collapses to a unimodal distribution when the system size increases, even for kinetic constant values that result in two distinct stable solutions in the deterministic Schlögl model. Using zero-information (ZI) closure scheme, an algorithm for solving chemical master equations, we compute stationary probability distributions for varying system sizes of the Schlögl model. With ZI-closure, system sizes can be studied that have been previously unattainable by stochastic simulation algorithms. We observe and quantify paradoxical discrepancies between stochastic and deterministic models and explain this behavior by postulating that the entropy of non-equilibrium steady states (NESS) is maximum.

## 1. Background

Chemical reaction kinetics have been canonically modeled with ordinary differential equations since the pronouncement of the law of mass action kinetics, 150 years ago [1]. This macroscopic, continuous-deterministic modeling formalism is appropriate at the thermodynamic limit, when the volume of the system and the numbers of molecules of reactants all tend to very large values.

Markov chain models can be used for chemical reactions away from the thermodynamic limit [2]. Models are then formulated in terms of discrete numbers of molecules for each of the chemical species present at time *t*. The system evolves stochastically, and the all-encompassing chemical master equation (CME) can model the probability distribution of the system being at a particular state at time *t* [3].

Kurtz [4,5] explored the relationship between stochastic and deterministic models when the macroscopic equations have a unique, asymptotically stable solution, and demonstrated that the deterministic model is the thermodynamic limit of the stochastic one.

However, when the ordinary differential equations admit more than one stable solutions, the two formalisms may give starkly different results for even simple, small chemical reaction models [6,7,8,9]. The Schlögl model is such a simple model [10], described as follows [11]:

$$3\;X\overset{k_{1}}{\underset{k_{2}^{\prime }}{⇌}}2\;X+A\;\;X\overset{k_{3}}{\underset{k_{4}^{\prime }}{⇌}}B.$$

This is a one-dimensional model. The only variable is the number of molecules *X*. Species *A* and *B* are present in the system with constant concentrations, arriving from separate external reservoirs. Setting $k_{2}=k_{2}^{\prime }\;\left[A\right]$ and $k_{4}=k_{4}^{\prime }\;\left[B\right]$, the model is simplified as

$$3\;X\overset{k_{1}}{\underset{k_{2}}{⇌}}2\;X\;\;X\overset{k_{3}}{\underset{k_{4}}{⇌}}\emptyset .$$

For a large system of volume *V*, the concentration x=X/V changes in time according to the law of mass action,

(1) $$\frac{dx}{dt}=-k_{1}\;x^{3}+k_{2}\;x^{2}-k_{3}\;x+k_{4}.$$

This cubic equation admits either a single stable solution, or two stable solutions and one unstable one [12]. The solution depends on kinetic constant and reservoir concentration values. Herein we retain values of all kinetic parameters constant, except $k_{4}$. The values of the constant kinetic parameters are $\left(k_{1},\;k_{2},\;k_{3}\right)=\left(0.42,\;70,\;3150\right)$. We present results for $k_{4}$ ranging from $10^{4}$ to $7\times 10^{4}$.

The chemical master equation for the Schlögl model has been formulated before [2,12,13]:

(2) $$\begin{array}{c} \frac{\partial }{\partial t}p\left(X;t\right)=\left[\frac{k_{2}}{V}\left(X-1\right)\left(X-2\right)+k_{4}V\right]p\left(X-1;t\right)\\ -\left[\frac{k_{2}}{V}X\left(X-1\right)+k_{4}V\right]p\left(X;t\right)\\ +\left[\frac{k_{1}}{V^{2}}\left(X+1\right)X\left(X-1\right)+k_{3}\left(X+1\right)\right]p\left(X+1;t\right)\\ -\left[\frac{k_{1}}{V^{2}}X\left(X-1\right)\left(X-2\right)+k_{3}X\right]p\left(X;t\right). \end{array}$$

As discussed previously [14], for small system sizes the stationary probability distribution p(X;t) is either unimodal or bimodal, depending on parameter values. Difficulties in computing the stationary probability distribution for mesoscopic systems, which are still under the influence of molecular fluctuations, but have molecular populations too large for stochastic simulation algorithms, have hampered the analysis of stationary probability distributions.

Herein we solve the master equation using ZI-closure scheme [14] for large system sizes that were previously unattainable with other methods. We keep the concentration *x* constant, while systematically increasing the system size, in order to investigate the collapse of bimodality for mesoscopic and large systems, and determine the limits of correspondence between CME and ODE models.

Please note that the systems studied in this work are not at equilibrium. The chemical potentials of the two reservoirs in the Schlögl reaction are set to be different. There is then mass flowing from the reservoir with high chemical potential to the reservoir with low chemical potential. The flow of mass is at steady state, and as a result the probability distribution of *X* within the system is stationary. By varying the kinetic constants, the steady state changes. We study the stationary probability distribution of these non-equilibrium steady states (NESS). NESS is one of three types of dynamics observed in chemical reaction systems [12]: (a) equilibrium state with fluctuations as described by classical statistical mechanics theories, (b) time-dependent, transient processes in which the state of systems changes with time, and (c) NESS [15].

We study the Schlögl model as a simple example of a bistable system. This model has been used extensively to model physical and chemical systems [16,17,18,19,20,21,22,23,24]. There is also a plethora of biological systems exhibiting bistability, such as the lysis and lysogeny system in phage [25]. Over the past two decades, there has been particular interest in both synthetic and natural biological systems exhibiting either temporal or spatial bistability [25,26,27,28,29,30]. The Schlögl model has been recognized as one of the simplest models that can capture essential elements of bistable behavior [31]. In principle, insights gained from studying this model can be applied to other bistable stochastic chemical reaction networks.

We also note that there a plethora of alternative, approximate numerical methods to solve stochastic reaction systems. The goal of the current manuscript is not to provide an exhaustive review of the literature, but we bring to the attention of the reader the following important references that describe Fokker-Planck, Linear-Noise and moment-closure approximations [32,33,34].

## 2. Zero-Information Closure Scheme

In this section, we briefly discuss the elements of the ZI-closure scheme. More details on the method can be found in [14,35,36].

Instead of attempting to solve directly the CME, an impossible task for all but the simplest of reaction networks, one can generate a set of ODEs that describe the time dynamics of the probability distribution moments [37,38]. The CME for the Schlögl model can be transformed in moment equations with the use of Z-transform of the probability distribution [38]:

(3) $$G\left(\Phi ,t\right)=\sum_{X=0}^{\infty }\Phi^{X}p\left(X,t\right),$$

where Φ is a new variable. Using derivatives of *G* w.r.t. Φ and *t*, the CME can be transformed into a set of moment equations:

(4) $$\frac{d\mu }{dt}=A\mu +A^{\prime }\mu^{\prime }+\mu_{0},$$

where μ is the vector of lower-order moments, $\mu^{\prime }$ is the vector of higher-order probability moments and $\mu_{0}$ a constant vector representing the zero-order moment.

Herein, moments are defined as expected values of the probability distribution:

(5) $$\left\{X^{m}\right\}=\sum_{X=0}^{\infty }\frac{X!}{(X-m)!}p\left(X,t\right),$$

where $\left\{X^{m}\right\}$ is the *m*th factorial moment of the probability distribution.

For example, the first four moment equations for the Schlögl are calculated as:

(6) $$\begin{array}{cc} \frac{d}{dt}\left[\begin{array}{c} \left\{X\right\}\\ \left\{X^{2}\right\}\\ \left\{X^{3}\right\}\\ \left\{X^{4}\right\} \end{array}\right] & =\left[\begin{array}{cccc} -k_{3} & \frac{k_{2}}{V} & -\frac{k_{1}}{V^{2}} & 0\\ 2k_{4}V & 4\frac{k_{2}}{V}-2k_{3} & 2\frac{k_{2}}{V}-4\frac{k_{1}}{V^{2}} & -2\frac{k_{1}}{V^{2}}\\ 0 & 6\frac{k_{2}}{V}+3k_{4}V & -6\frac{k_{1}}{V^{2}}-3k_{3} & 3\frac{k_{2}}{V}-12k_{4}V\\ 0 & 0 & 24\frac{k_{2}}{V}+4k_{4}V & 24\frac{k_{2}}{V}-24\frac{k_{1}}{V^{2}}-4k_{3} \end{array}\right]\left[\begin{array}{c} \left\{X\right\}\\ \left\{X^{2}\right\}\\ \left\{X^{3}\right\}\\ \left\{X^{4}\right\} \end{array}\right]\\ & +\left[\begin{array}{cc} 0 & 0\\ 0 & 0\\ -3\frac{k_{1}}{V^{2}} & 0\\ 4\frac{k_{2}}{V}-24\frac{k_{1}}{V^{2}} & -4\frac{k_{1}}{V^{2}} \end{array}\right]\left[\begin{array}{c} \left\{X^{5}\right\}\\ \left\{X^{6}\right\} \end{array}\right]+\left[\begin{array}{c} k_{4}V\\ 0\\ 0\\ 0 \end{array}\right] \end{array}$$

For the Schlögl model, we have empirically found that at least ten moments are needed to accurately capture the probability distribution, especially when it is bimodal [37,38,39].

The dependence of the lower-order vector $\mu ={\left[\left\{X\right\}\;\left\{X^{2}\right\}\;\left\{X^{3}\right\}\;\left\{X^{4}\right\}\right]}^{T}$ on the higher-order one $\mu^{\prime }={\left[\left\{X^{5}\right\}\;\left\{X^{6}\right\}\right]}^{T}$ is evident in this equation. This is the closure scheme challenge, which we have previously solved by developing the ZI-closure scheme [14,35,36].

For a steady state, the left-hand side of the moment equation is zero. To solve these equations, we postulate that the probability distribution attained by the system is the one that maximizes the entropy [40], which is given by the equation:

(7) $$S=-\sum_{X}p\left(X\right)\ln p\left(X\right).$$

Using the method of Lagrange multipliers, the probability distribution p(X) can then be expressed as:

(8) $$p\left(X\right)=\exp\left[-\sum_{i=0}^{\Psi }\lambda_{i}\frac{X!}{(X-i)!}\right],$$

where $\lambda_{i}$ is the Lagrange multiplier associated with the lower-order moment $\mu_{i}$ and Ψ represents the size of vector μ.

The moments are related to Lagrange multipliers through Equations (5) and (8). Consequently, an important feature of the ZI-closure scheme algorithm is that Equation (6) depends only on the Lagrange multipliers. For a finite, explicitly defined state space, the sums in Equation (7) can be considered explicitly. We then use a root-finding method (e.g., Newton-Raphson) to calculate the Lagrange multipliers. The stationary probability distribution is finally directly calculated [14].

An advantage of ZI-closure over stochastic simulation algorithms is that it calculates steady state probability distributions directly, without resorting to simulations in time. Stochastic simulations must start with a specific initial condition in time for an ensemble of trajectories. Each trajectory can eventually reach a steady state. Instead, ZI-closure algorithm is initiated with a specific initial guess for the stationary probability distribution (e.g., a delta function) and numerically converges to the steady-state p(X).

## 3. Results

In this section we present results obtained with ZI-closure scheme for the stochastic Schlögl model.

### 3.1. Bimodality Collapse for Mesoscopic Systems

**Figure 1:**
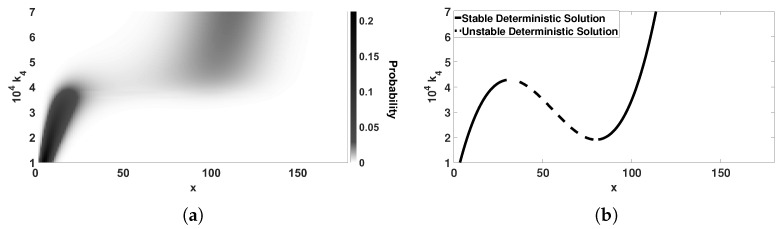
The figure shows the effect of kinetic constant $k_{4}$ on the stationary probability distribution for small systems with V=1. (**a**) shows the behavior of the stochastic system. Darker areas present higher probability. As the kinetic constant increases, the system transitions from a unimodal distribution to a bimodal and then back to a unimodal one. (**b**) shows the behavior of the deterministic system. Solid lines represent the stable deterministic solutions and the dashed-dotted line represents the unstable solution of the ODE model. The other kinetic constants are: $\left(k_{1},\;k_{2},\;k_{3}\right)=\left(0.42,\;70,\;3150\right)$.

In Figure 1a, steady state probability distributions, p(x), are plotted for a range of values of $k_{4}$.

In accordance with the conclusion drawn by Kurtz, when the probability distribution is unimodal, the average of *X* corresponds to the single stable ODE solution. This is shown in the Figure 1b, where the ODE solution has been plotted on the *x*-$k_{4}$ plane. We also observe that when the distribution is bimodal, the peaks correspond to the two stable deterministic attractors (Figure 1). A main purpose of this article is to explore deviations from this congruency, as the system size varies.

In the rest of the document, the terms “probability peak (peak)” and “attractor” will be used interchangeably. When the intention is to draw attention to the probability distribution and focus on the stochastic behavior the term “peak” is preferable. On the other hand, when the intention is to compare the results of the deterministic and stochastic models, we will prefer the term “attractor”, which pertains to both modeling formalisms.

Parenthetically, it is interesting to note that the range of $k_{4}$ values where the stochastic model exhibits bimodality does not precisely correspond to the range of $k_{4}$ values where the deterministic model exhibits bistability. The reason for this minor discrepancy is that in the ODE model (Equation (1)) higher order reactions are represented in terms of $X^{2}$ and $X^{3}$, whereas in the CME (Equation (2)), there are X(X−1), (X−1)(X−2), (X+1)X(X−1) and X(X−1)(X−2) terms.

When the system is bistable, only one of the solutions will be reached in finite time in the ODE model. Which one of the solutions will be reached depends on the initial conditions. In contrast, the stochastic model is ergodic and explores the entire state space (all the possible numbers of molecules of *X*) with a frequency proportional to the probability, visiting both attractors, regardless of initial conditions.

In other words, the stochastic model identifies and distinguishes all solution attractors, whereas the ODE model cannot. This is in accord with, among others, the study of Ge and Qian [41], who concluded that although deterministic differential equations can define numerous attractors, they provide no information on the relative probabilities between them. Only stochastic model solutions can provide such insight.

What is intriguing is that as the system size increases, the stochastic Schlögl model behavior ceases to correspond to the ODE one. The deterministic model solution is not dependent on the size of the system and will always exhibit the same bistable behavior, for certain ranges of parameter values. In the stochastic model, the size of the system plays a critical role. As the size increases, the bimodality can be destroyed, at least in numerical terms, and the stationary probability distribution can become a delta-like function, as observed previously [6,7,9,10,42,43,44].

**Figure 2:**
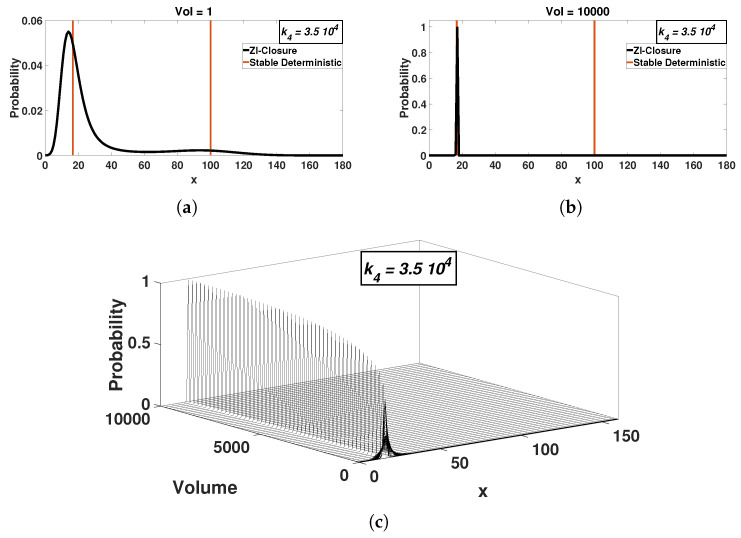
The probability distributions are shown for different volumes, for $k_{4}=3.5\times 10^{4}$ (**c**). The system starts with bimodal distributions at small volumes (**a**). Bimodality collapses as the system size increases. The stochastic model attains only one of the two deterministic solutions as the volumes increases (**b**). The solid vertical lines represent the stable deterministic solutions. (**c**) has 220 plotted probability distributions computed with ZI-closure.

**Figure 3:**
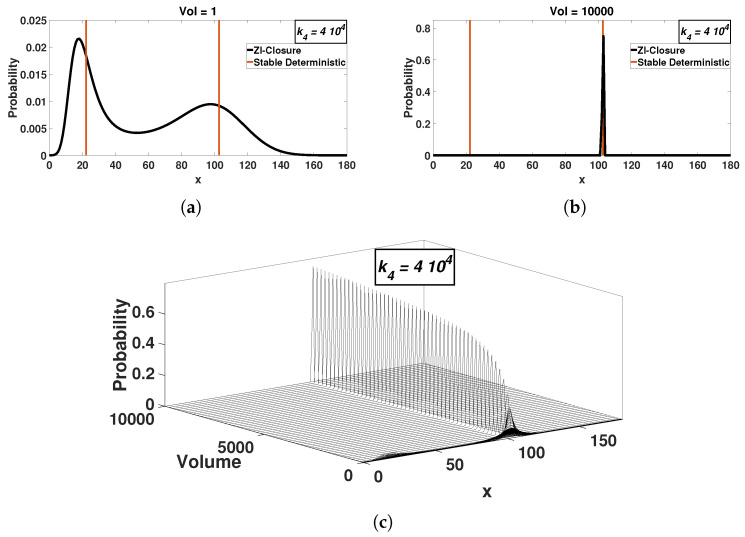
Similar results to Figure 2, however here $k_{4}=4.0\times 10^{4}$. Bimodality collapses as the system size increases as well (**a**). However in this case, the stochastic model attains a different deterministic attractor as the volumes increases (**b**) compared to Figure 2. The solid vertical lines represent the stable deterministic solutions and (**c**) has 220 plotted probability distributions computed with ZI-closure.

This behavior is observed in Figure 2 and Figure 3, where the stationary probability distribution is shown as a function of the volume for two values of $k_{4}$. Both $k_{4}$ values are within the range that produces two stable solutions for Equation (1). For small volumes, the stochastic model stationary probability distribution exhibits bimodality. The peaks then approximately correspond to the ODE solutions (Figure 2a and Figure 3a). We note again that this type of behavior was observed before, e.g., in [9].

As the volume increases, the model’s bimodality apparently disappears, with only one peak remaining significant (in numerical terms, the size of the second peak is lower than the numerical, computer accuracy). The disappearance of bimodality occurs gradually with the system size, as one of the peaks becomes progressively smaller. Even though the less dominant peak might still exist in finite volumes, we find that peak sizes fall below computer round-off errors. We speculate then that at the thermodynamic limit only one peak survives. Not unexpectedly, as the volume increases, probability distribution standard deviations decrease, resulting in a delta function centered at one of the ODE solutions (Figure 2b and Figure 3b). In other words for large system sizes, the stochastic model reaches only one solution whereas the ODE model can have three solutions, two stable and one unstable.

Because of system size models previously unattainable with stochastic simulations and because of the unexpected results, we have taken pains in ensuring the convergence and accuracy of the ZI-closure scheme results presented herein. The reader is directed to the appendix for more information about the accuracy of ZI-closure results. There are three ways to increase confidence in ZI-closure results:

First, in principle, for stochastic systems that satisfy the ergodic hypothesis, the CME solution does not depend on initial conditions. In practice, we have validated this hypothesis by determining CME solutions with the ZI-closure scheme, numerically starting with various initial probability distribution solutions (prescribed in the initial values of the Lagrange multipliers in the numerical scheme).

Second, ZI-closure solutions of the CME are verified by the analytical expressions available for the Schlögl model [2,43]. We note that even though the analytical solution is available, the numerical implementation becomes impractical for large volumes because of numerical range limitations in the calculation of the probability at X=0 (Appendix A). For the kinetic constant of Figure 2 ($k_{4}=3.5\times 10^{4}$) and Figure 3 ($k_{4}=4\times 10^{4}$), the analytical solution fails to produce results for volume values larger than V=58 and V=45, respectively.

Finally, ZI-closure solutions is verified by comparison to the probability distribution obtained from stochastic simulation algorithm (SSA) simulations [45].

**Figure 4:**
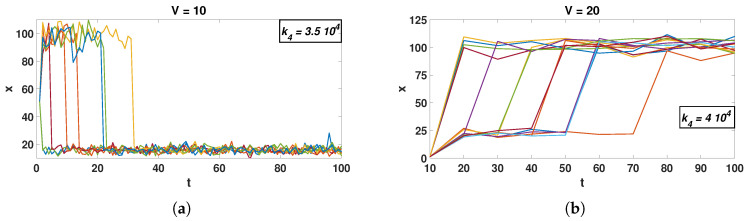
Sample SSA trajectories for two different kinetic constants ((**a**): $k_{4}=3.5\times 10^{4}$ and (**b**): $k_{4}=4\times 10^{4}$). Even though trajectories start from a different attractor, they transition to the stochastic stationary one. As in Figure 1, *x* is the concentration X/V. For visual clarity, the figure presents only a random sample of 500,000 simulated trajectories, all of which behave in a similar fashion.

In light of the bimodality collapse results, it is interesting to conduct SSA simulations with trajectories that start at one of the ODE solutions. We observe all trajectories moving to the attractor that is attained by ZI-closure, regardless of initial conditions. Surprisingly, system trajectories quickly transition to the stationary attractor, even if they are initiated at the second attractor (Figure 4). This behavior is counterintuitive, especially in light of the deterministic model solutions, where the steady state is attained that is closest to the initial conditions of a simulation.

A quantity that has been used to explain the collapse of bimodality in the Schlögl probability distribution for mesoscopic systems is the passage or transition time. Mean (first) passage times can be calculated in a single-step, one-dimensional Markov chain with a bimodal distribution. They represent the time that is required for the system to move from one peak to the other and can be calculated from the following formulas [2]:

(9) $$T_{ac}=\sum_{x=a}^{c-1}\frac{1}{W_{+}\left(x\right)p\left(x\right)}\sum_{y=0}^{x}p\left(y\right)\;T_{ca}=\sum_{x=a+1}^{c}\frac{1}{W_{-}\left(x\right)p\left(x\right)}\sum_{y=x}^{\infty }p\left(y\right),$$

where, $T_{ac}$ is the mean passage time for moving from the left peak (*a*) to the right (*c*) and $T_{ca}$ the time for moving from the right to the left, at steady state. $W_{+}\left(X\right)$$=\frac{k_{2}}{V}\left(X\right)\left(X-1\right)+k_{4}V$ and $W_{-}\left(X\right)$$=\frac{k_{1}}{V^{2}}X\left(X-1\right)\left(X-2\right)+k_{3}X$ are the transition rates.

**Figure 5:**
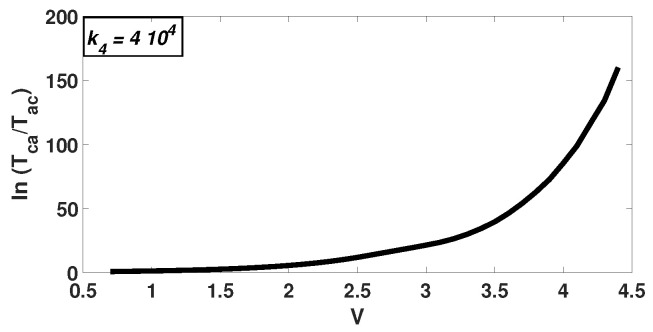
The figure shows the ratio of the time required to transition from the right peak to the left one over the time to move from the left to the right. This ratio increases exponentially with the system volume at steady state. In this figure, results for only one kinetic constant value ($k_{4}=4\times 10^{4}$) are presented.

By calculating the mean passage time for different volumes (Figure 5), it is observed that the time moving from the right peak to the left over the time that it takes from left to right increases exponentially. The results support previous findings [8]. Even though a second peak might exist in higher volumes, it is significantly less important than the dominant one. Again, this implies that only one peak may be recovered at the thermodynamic limit.

### 3.2. Entropy of NESS Systems

ZI-closure postulates that a stochastic reaction system reaches the stationary probability distribution that maximizes the system’s entropy.

We next study how the entropy of NESS systems changes with the volume and with $k_{4}$. We vary *V* and $k_{4}$ and compute the stationary distribution for each set of parameters. Each NESS has a single entropy value.

**Figure 6:**
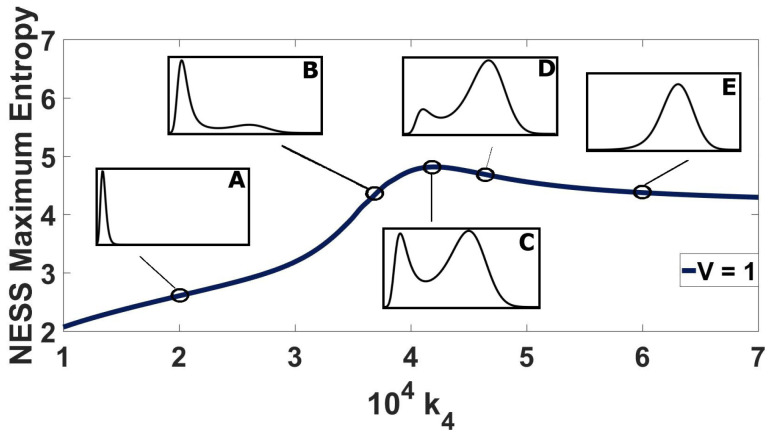
NESS entropy for varying kinetic constant ($k_{4}$) values at V=1. Example stationary probability distributions are depicted, as insets, to indicate the five distinct trends of NESS entropy with $k_{4}$. The corresponding kinetic values are: $k_{4}=2\times 10^{4}$ (**A**), $k_{4}=3.7\times 10^{4}$ (**B**), $k_{4}=4.21\times 10^{4}$ (**C**), $k_{4}=4.5\times 10^{4}$ (**D**) and $k_{4}=6\times 10^{4}$ (**E**).

In Figure 6, the entropy of computed NESS is plotted for 600 different values of $k_{4}$ at volume V=1. Please note that with so many points, there is the appearance of a continuous line in the graph.

At small kinetic constant values, the stationary probability distribution is unimodal (Figure 6A), with the peak being centered at the sole stable attractor of the deterministic model, located on the left-hand side of the state space. In this region of $k_{4}$ values, the entropy of the system increases linearly with increasing $k_{4}$.

At a certain value of $k_{4}$, the ODE will yield three solutions. The stationary probability distribution becomes bimodal at that point, with the peak on the left attractor being dominant. The entropy of bimodal NESS continues increasing linearly with $k_{4}$, albeit with a larger slope.

At a critical $k_{4}$ value, NESS entropy reaches a maximum. At this critical value, the stationary distribution peaks have approximately the same probability (Figure 6C).

As $k_{4}$ is increased more, the probability distribution maintains its bimodal shape, but with the right peak dominating (Figure 6D). The entropy of the system begins to decrease with $k_{4}$. Again, at a certain value of $k_{4}$, the probability distribution becomes unimodal (Figure 6E), with the peak located at the right hand side of the state space, resulting in another slope change.

**Figure 7:**
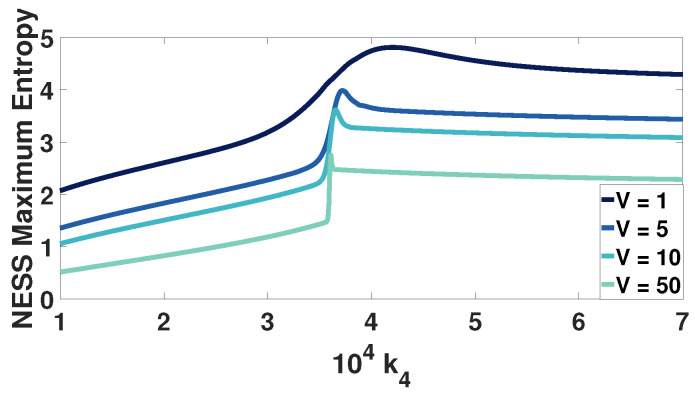
The maximum entropy of different NESS probability distributions plotted against the kinetic constant $k_{4}$, for four different volumes *V*. Each volume line includes more than 600 points, with each one of them corresponding to a different NESS. With so many points there is the appearance of a continuous line for each *V*.

In Figure 7, we present the NESS entropy as a function of $k_{4}$ for four different system sizes, varying the volume by 50. Again 600 NESS probability distributions are computed for different $k_{4}$ values at each volume, and their entropies are plotted in this graph.

The bimodal region of the system for each volume is located between the two slope changes. As the volume increases, the range of $k_{4}$ values resulting in bimodal stationary probability distribution decreases. This leads us to speculate that at the thermodynamic limit, the bimodal region may consist of a single critical $k_{4}$ value. At this point, the probability distribution will have two equally weighted, distinct peaks with a total entropy of −$\ln\left(0.5\right)$.

## 4. Discussion

The differences between deterministic and stochastic models for chemical reaction networks have long been the subject of numerous investigations [6,7,8]. Herein we also present results that highlight such differences, focusing on the collapse of bimodality of the Schlögl model for mesoscopic and macroscopic systems.

The size of the system is an important parameter. For small systems, the stochastic model produces a bimodal stationary distribution with peaks near the ODE solutions. As the system size increases, the stochastic model gradually prefers only one of the two attractors. Please note that the observed destruction of bimodality is solely a numerical interpretation of the results. In other words, we observe the one peak disappearing only in the numerical sense. It may well be the case that a second peak is present but at a value lower than the allowable computer precision. We observe this behavior with ZI-closure scheme solutions, with analytical solutions and with SSA trajectories.

The question then arises: what drives a mesoscopic system to only one of the two attractors? This is especially puzzling, when the initial condition is the second attractor and both of these attractors are stable solutions of the ODE model.

Numerous explanations have been provided for the discrepancies, including ones based on the mean transition times between peaks [8]. In other studies, efforts were made to connect these differences to the entropy production rate [41,43]. In fact, numerous efforts have attempted, and ultimately failed, to establish whether a stability criterion for non-equilibrium steady states can exist based on the rate of entropy production [43,46,47,48,49,50]. We remind the reader that when closed systems are at an equilibrium state the stability criterion is that the entropy is maximum. There is no such established criterion for non-equilibrium steady states.

In this study, NESS stationary probability distributions are reached when the entropy of the system is maximum. We stress that the ZI-closure scheme postulates a maximum entropy for every NESS and then computes the correct probability distribution, numerically matching the ones from kinetic Monte Carlo simulations and the analytical solution. In other words, the solution of the CME obtained by ZI-closure scheme is the probability distribution with maximum entropy. This was also observed previously for numerous other stochastic chemical reaction systems, including stochastic dimerization, stochastic Michaelis-Menten and stochastic cycle reaction networks [14].

Please note that the total entropy of the system and the two reservoirs increases with time. Given a non-zero difference between the chemical potentials of the two reservoirs, there is mass transfer from the high to the low chemical potential reservoir. This is overall an irreversible process with positive entropy production. Yet, within the system of interest, a stationary probability distribution is attained, which implies that the entropy of the system contained within volume *V* is constant. Additionally, for open, isothermal, isochoric systems, the relevant equilibrium thermodynamic property is the Helmholtz free energy. Herein the defined Schlögl model is void of enthalpic constraints. Thus a minimum Helmholtz free energy is equivalent to a maximum entropy.

## 5. Summary

We present solutions of the CME for Schlögl model systems. The results cover a wide range of system sizes, previously unattainable with traditional methods (e.g., SSA, analytical solution). This is possible now with the employment of ZI-closure scheme. As reported previously, for mesoscopic systems, the exhibited bimodality collapses and only one of the attractors becomes dominant with probability 1.

It is revealed that the range of kinetic constant values where bimodality survives, at least in numerical terms, diminishes in larger systems. Presumably, there is a single value of $k_{4}$ where bimodality apparently survives at the thermodynamic limit. This is only speculative because, although the ZI-closure scheme affords the investigation of system sizes that remained inaccessible in previous studies, it is also still limited to finite volumes.

The ZI-closure scheme postulates a maximum entropy for non-equilibrium steady states in order to numerically close the moment equations and compute the stationary probability distribution. We wonder whether one can argue that since the probability obtained is the correct one, it follows that the entropy of this NESS is maximum. In other words, at a stable NESS the system will attain values for the number of molecules of *X* that result in the probability distribution with a maximum entropy. We will continue investigating whether this criterion holds generally for NESS systems.

## Figures and Tables

**Figure A1:**
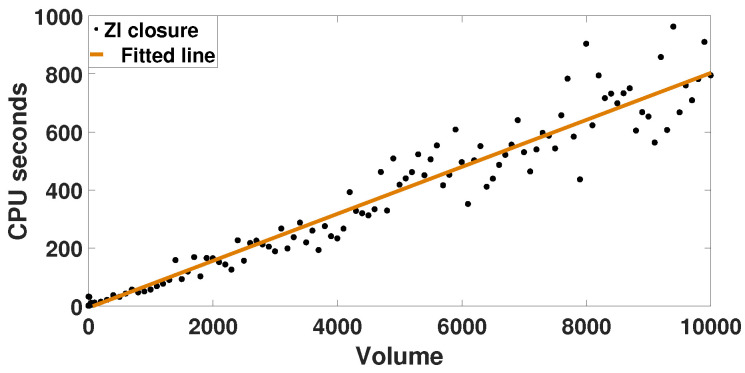
CPU time required to run ZI-closure scheme for different volumes. The computational running time of ZI-closure scheme scales linearly with system’s size. In this system $k_{4}=4\times 10^{4}$.

**Figure A2:**
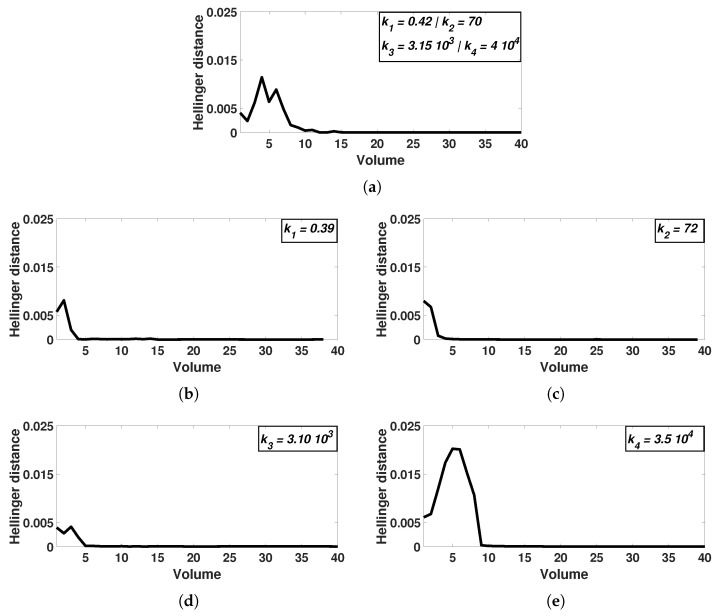
Difference between probability distributions calculated with analytical solution and ZI-closure. The kinetic constants of (**a**) are shown in each caption. For (**b**)–(**e**), each plot has only one different kinetic constant compared to (**a**). The values of the kinetic constants that are different for each figure can be found on the top right corner. The plots represent solutions for a variety of volumes. The accuracy of ZI-closure scheme is not affected by system size. For this comparison, the Hellinger distance was used [52].

## Appendix A. ZI-Closure Accuracy

The most popular method to solve stochastic reaction networks is SSA [45]. Even though SSA is accurate, it is computationally expensive. As a result, SSA is often impractical for parametric analysis. For example, for the Schlögl model for the kinetic parameters reported earlier and volume V=3, SSA needs more than 1500 CPU hours to reach steady state for a simulation with 10${}^{5}$ trajectories. In comparison, ZI-closure scheme for the same system needs only 2 CPU seconds. We have previously presented comparisons between ZI-closure and SSA for several chemical reaction networks, including Schlögl [35,51].

ZI-closure scheme is effectively faster because it directly calculates the steady state distribution without calculating the transient behavior of the system. Additionally, the CPU time required by ZI-closure scheme scales linearly with volume (Figure A1).

Another way to calculate steady state distributions for the Schlögl reaction network is through the analytical solution [43]:

(A1) $$\frac{p(X)}{p(0)}=\prod_{n=0}^{X-1}\frac{\frac{k_{2}}{V}\left(n\right)\left(n-1\right)+k_{4}V}{\frac{k_{1}}{V^{2}}\left(n+1\right)n\left(n-1\right)+k_{3}\left(n+1\right)}\;,\;p\left(0\right)=1-\sum_{k=0}^{\infty }p\left(k\right)$$

where p(X) is the stationary probability at the state space point *X*. p(0) is the probability at state space point X=0. This equation can be easily derived from CME if the probability flux in the forward direction is set equal to the probability flux in the backwards direction at each *X* at steady state (detailed balance condition).

The analytical expression fails to produce results for large volume values, without approximations that may impact the accuracy. This is because the probability p(0) goes to zero very fast as the system size increases.

We investigate volume sizes that reach in the thousands. The largest number Matlab can handlex is $1.7977\times 10^{308}$, so results quickly get out of the numerical precision range of modern computers. To our knowledge, there is no way to compute the solution using the analytical expression for large volumes, given the kinetic constant values we employ. For example, for kinetic constant value $k_{4}=4.0\times 10^{4}$, volume V=44 is the last one the analytical solution produces results (Table A1).

**Table A1:** Representative values are shown (second row) of the stationary probability distribution at X=0 (p(0)) at different volumes (first row). The table also includes the ratio of the maximum value of the stationary probability distribution over p(0). The values are obtained for $k_{4}=4.0\times 10^{4}$.

Volume	1	10	20	30	40	44
p(0)	$1.89\times 10^{-7}$	$4.14\times 10^{-70}$	$4.17\times 10^{-140}$	$4.18\times 10^{-210}$	$4.18\times 10^{-280}$	$4.19\times 10^{-308}$
$\frac{\mathrm{maximum}\;p(X)}{p(0)}$	$1.14\times 10^{5}$	$1.54\times 10^{67}$	$1.09\times 10^{137}$	$8.91\times 10^{206}$	$7.72\times 10^{276}$	$7.36\times 10^{304}$

Here also lies the significance of our approach. We use a probabilistic modeling formalism to study systems with sizes that span a vast range, practically tending to the thermodynamic limit and can thus investigate the transition between stochastic and deterministic dynamics in chemical reaction systems.

Importantly, ZI-closure scheme is as accurate as the analytical solution, as observed in Figure A2. Figure A2 presents the effect of the volume size on the accuracy of ZI-closure scheme for multiple kinetic constants. For every kinetic constant, the probability distribution at Volume V=1 is bimodal. The accuracy of the method is not affected as the system size increases (Figure A2).
